# Artificial intelligence for instance segmentation of MRI: advancing efficiency and safety in laparoscopic myomectomy of broad ligament fibroids

**DOI:** 10.3389/fonc.2025.1549803

**Published:** 2025-04-08

**Authors:** Feiran Liu, Minghuang Chen, Haixia Pan, Bin Li, Wenpei Bai

**Affiliations:** ^1^ Department of Obstetrics and Gynecology, Beijing Shijitan Hospital, Capital Medical University, Beijing, China; ^2^ College of Software, Beihang University, Beijing, China; ^3^ Department of MRI, Beijing Shijitan Hospital, Capital Medical University, Beijing, China

**Keywords:** artificial intelligence - AI, uterine myoma, Instance segmentation, laproscopic myomectomy, MRI

## Abstract

**Background:**

Uterine broad ligament fibroids present unique surgical challenges due to their proximity to vital pelvic structures. This study aimed to evaluate artificial intelligence (AI)-guided MRI instance segmentation for optimizing laparoscopic myomectomy outcomes.

**Methods:**

In this trial, 120 patients with MRI-confirmed broad ligament fibroids were allocated to either AI-assisted group (n=60) or conventional MRI group (n=60). A deep learning model was developed to segment fibroids, uterine walls, and uterine cavity from preoperative MRI.

**Result:**

Compared to conventional MRI guidance, AI assistance significantly reduced operative time (118 [112.25-125.00] vs. 140 [115.75-160.75] minutes; p<0.001). The AI group also demonstrated lower intraoperative blood loss (50 [50-100] vs. 85 [50-100] ml; p=0.01) and faster postoperative recovery (first flatus within 24 hours: (15[25.00%] vs. 29[48.33%], p=0.01).

**Conclusion:**

This multidisciplinary AI system enhances surgical precision through millimeter-level anatomical delineation, demonstrating transformative potential for complex gynecologic oncology procedures. Clinical adoption of this approach could reduce intraoperative blood loss and iatrogenic complications, thereby promoting postoperative recovery.

## 1 Introduction

Uterine fibroids are the most prevalent benign tumors affecting the female reproductive system among child-bearing aged women. The morbidity rate exceeds 70%, significantly impacting female reproductive health (1). The manifestation of symptoms, including abnormal uterine bleeding, infertility, pelvic pain, and compression-related symptoms, is a key determinant in treatment approaches, which are closely tied to the size, quantity, and position of the fibroids (2). Consequently, surgical strategies are modified to align with these parameters. Generally, uterine fibroids are commonly intramural, submucosal, or subserosal; however, broad ligament fibroids, which are considered a diagnostic and surgical dilemma due to their unique anatomical location, present many challenges in clinical practice. Myomectomy for broad ligament fibroids is often complicated by surgical risks such as ureteric and uterine vessel injuries.

As patients suffered from uterine fibroids often lean towards minimally invasive procedures, laparoscopic myomectomy(LM) emerging as the primary surgical choice following its initial performance in the 1970s. The majority of FIGO uterine fibroid types can be removed through laparoscopic myomectomy (LM), including broad ligaments fibroids, which has demonstrated notable advantages compared to open myomectomy, including reduced postoperative pain, lower rates of postoperative fever, and shorter hospital stays (3). However, the anatomical complexity of broad ligament fibroids—particularly their proximity to uterine myometrium and retroperitoneal neurovascular bundles—introduces unique intraoperative risks that partially offset these benefits. Broad ligament fibroids present unique surgical challenges due to their embryological origin in the Müllerian duct remnants, which predispose them to several complications. These include: 1) interdigitation with uterine vascular arcades; 2) compression of the ureteric tunnel and 3) adherent peritoneal reflections that require precise dissection planes. Nonetheless, managing blood loss remains a significant challenge in laparoscopic myomectomy (LM). Zaki Sleiman et al. highlighted a correlation between blood loss during LM and factors such as the size and number of fibroids, as well as operative time, while excluding variables like age, body mass index (BMI), and menstrual cycle phase (4). Given that the size and quantity of fibroids are unmodifiable, streamlining operative time stands out as a potential breakthrough option. Besides that, despite technological advancements, laparoscopic management of myomectomy remains surgically demanding due to three inherent challenges: (1) Restricted visual field limitations imposed by the retroperitoneal anatomy complicate intraoperative orientation, increasing risks of ureteral injury (2) The intimate proximity of fibroids to uterine myometrium and parametrial plexus predisposes to catastrophic hemorrhage when conventional 2D imaging guidance is used (3) Conventional MRI reconstruction techniques lack dynamic spatial correlation with real-time laparoscopy, resulting in suboptimal surgical planning.

Precisely targeting this temporal challenge, Artificial Intelligence (AI) has increasingly extensive application in surgical interventions to enhance both efficiency and safety. Pietro Mascagni et al. pioneered the development of a deep learning model aimed at automating the segmentation of hepatocystic anatomy during laparoscopic cholecystectomy (5). In the realm of gynecological surgery, Sabrina Madad Zadeh et al. curated two datasets comprising laparoscopic gynecological images and crafted an artificial neural network for semantic segmentation specifically tailored for laparoscopic images during gynecological procedures (6, 7). Furthermore, they integrated augmented reality into LM guidance, albeit with reservations regarding its clinical implementation (7–9). It ‘s worth noting that the aforementioned augmented reality approach still necessitates the involvement of a radiologist to perform the segmentation of the uterus and fibroids, constructing a three-dimensional (3D) mesh model using preoperative magnetic resonance (MR) images. In a recent study by Yoshifumi Ochi et al., mixed reality was employed in a singular patient during LM; however, the challenge of relying on preoperative MR images for segmentation still persists (10). In summary, for broad ligament LM, AI-driven MRI segmentation directly addresses the operative time-blood loss paradigm through three mechanisms: (1) Preoperative 3D reconstruction of fibroid-myometrium interfaces reduces intraoperative anatomical exploration time (2) Automated quantification of fibroid spatial distribution enables optimized trocar placement strategies, minimizing instrument repositioning delays; (3) Real-time AI-enhanced visualization compensates for the lack of tactile feedback in laparoscopy, particularly crucial when dissecting parametrial adhesions.

Image segmentation has emerged as a pivotal component in the application of deep learning methodologies within the domain of medical AI. Yasuhisa Kurata et al. employed U-net and adjusted parameters to achieve automatic segmentation of the uterus in MRI images (11). This segmentation algorithm underwent rigorous testing on MR T2-weighted sagittal images encompassing conditions such as uterine cervical cancer, endometrial cancer, and uterine fibroids. Alireza Fallahi et al. introduced the Fuzzy C-Mean algorithm along with morphological operations, demonstrating successful automatic segmentation on MR T1-weighted sagittal images (12). Addressing the segmentation of uterine fibroids on MR images, Jian Zhang et al. proposed a modified U-Net with integrated attention mechanisms focusing on both channel and spatial aspects (13).

In the treatment of uterine fibroid, researchers have incrementally applied AI-driven automatic segmentation to High-Intensity Focused Ultrasound (HIFU) treatment.

Carmelo Militello et al. innovatively proposed algorithms based on Fuzzy C-Means clustering and iterative optimal threshold selection (14). This method autonomously segmented MR images during HIFU treatment in fibroid patients. Similarly, Kari Antila et al. developed an algorithm for automatic segmentation specifically designed for promptly detecting uterine fibroid regions following MR-guided High-Intensity Focused Ultrasound treatment (15). However, HIFU treatment still remains some limitations, as comparing to the surgery, which has greater recurrence rate and indefinite following pregnancy outcomes. Consequently, the first line treatment of uterine fibroids is still resection.

To the best of our knowledge, there is currently no existing research exploring the application of AI segmentation to assist LM. The majority of contemporary segmentation algorithms have predominantly centered around semantic segmentation of the uterus, posing prominent limitations for LM. In response to this gap, our team undertook the construction of a comprehensive uterine fibroid MR dataset, encompassing all FIGO types and comprising data from 300 fibroid patients. Furthermore, we pioneered the development of instance segmentation algorithms rooted in deep learning, which significantly enhance fibroid detection and classification (16). This method involved the optimization of the Mask-RCNN model, a crucial benchmark in numerous instance segmentation algorithms. Our algorithms demonstrate the capability to achieve precise instance segmentation of fibroids, uterine walls, and cavities, thereby facilitating high-quality surgical decision-making. While differential diagnosis from uterine sarcomas remains critical in fibroid management, the current AI model focuses on surgical precision enhancement rather than malignancy prediction—a direction we are actively pursuing in parallel investigations. Future iterations may incorporate sarcoma risk stratification by analyzing interface texture features.

This paper marks the inaugural introduction of AI automatic segmentation on MR images into the realm of preoperative planning for LM of broad ligament fibroids. Gynecologists now possess enhanced capabilities for strategic decision-making in terms of selecting optimal surgical incisions and determining the spatial location of fibroids. As a result, patients undergoing AI-assisted procedures experienced reduced operation duration, diminished blood loss, and a shortened timeframe to achieve the first postoperative flatus. These outcomes underscore the huge potential of AI in advancing the field of gynecologic laparoscopic surgery.

## 2 Methods

### 2.1 Participants and study design

Participants in this study were enrolled from July 2022 to November 2023 at Beijing Shijitan Hospital. A total of 120 patients with broad ligament fibroids were included, with age ranging from 24 to 44 years and fibroid size ranging from 4.00 to 10.67 cm. This study was conducted in accordance with the World Medical Association ‘s Declaration of Helsinki. And it was approved by the scientific research ethics committee of Beijing Shijitan Hospital, Capital Medical University [code: SJTKYLL-LX-2022(01)]. This study would not violate the rights and interests of patients. The ethics committee clearly stated that specific consent procedures were not required for this study.

Participants met the following inclusion criteria: 1.Symptomatic presentation requiring surgical intervention: Abnormal uterine bleeding (defined as menstrual volume >80 mL/cycle or duration >7 days) with hemoglobin <110 g/L. Compression symptoms (e.g., urinary frequency, hydronephrosis, or bowel dysfunction) confirmed by MRI. 2.MRI-confirmed broad ligament fibroids. 3.Postoperative pathological confirmation of benign leiomyoma. 4.High-quality preoperative MRI including T2-weighted axial sequences (slice thickness ≤3 mm) and diffusion-weighted imaging (b-value = 800 s/mm²) to ensure AI segmentation feasibility.

The exclusion criteria were as follows: 1. Severe comorbidities (ASA class ≥III) that independently affect surgical outcomes (e.g., uncontrolled heart failure, Child-Pugh C cirrhosis). 2.Active pelvic inflammation (CRP >10 mg/L AND body temperature >37.5°C). 3.Uterine active massive bleeding, severe anemia. 4.Pregnancy or lactation (serum β-hCG-positive). 5.Genital tuberculosis without anti-tuberculosis treatment. 6.Non-fibroid pathology on postoperative histology (e.g., adenomyosis, sarcoma).7.history of uterine perforation within 3 months.8.invasive cervical cancer. 9.with MRI contraindications, such as febrile convulsions, active foreign bodies in the eyes, cardiac pacemakers, metal intrauterine devices, metal joints and metal dentures. 10.Poor MRI image quality (motion artifact score ≥3 on a 5-point scale) precluding reliable AI segmentation.

**Figure 1:**
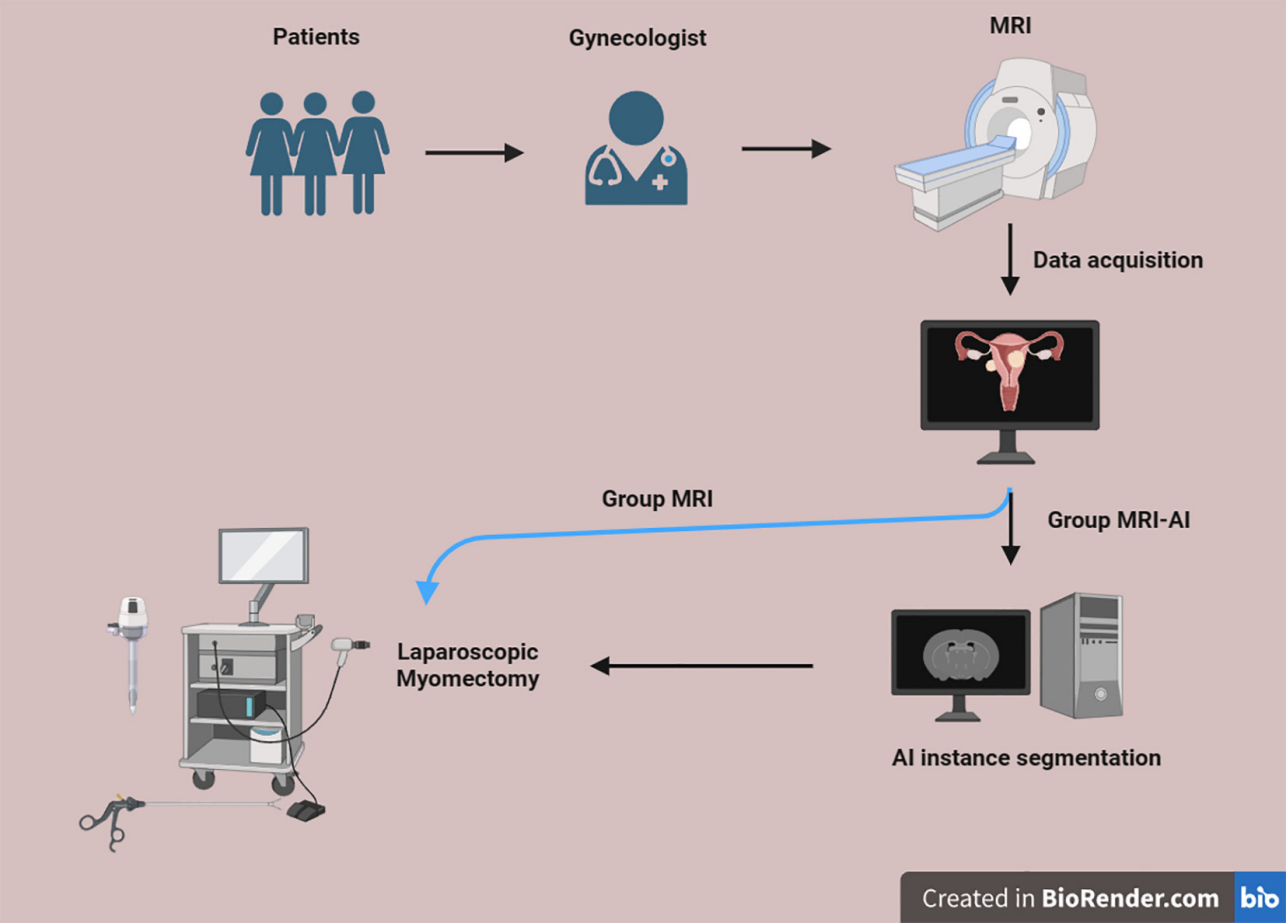
Flow chart.

This research was conducted according to the following process (**Figure 1**). All eligible subjects underwent MRI examination. Using a computer-generated random number table, eligible participants were equally allocated to either the MRI-artificial intelligence (MRI-AI) group (n=60) or the MR group (n=60). Half of them were divided into group MRI-AI, and the other half were divided into group MR. The surgical procedure in both groups was performed by the same surgeon, using the same instrument set, with abundant experience and the same surgical equipment, which is blinded to the group allocation.

### 2.2 MRI image acquisition

MRI examination in this study was completed in the PHILIPS INGENIA magnetic resonance imaging system with 3.0T ultra-high field. The MRI scan parameters were as follows: repetition time 4200ms, echo time 130ms, voxel 0.8x0.8x4.0cm3, field of view 24x24cm, reverse angle 90°. MRI provided multiple images from the sagittal, coronal and axial scans and from various sequences including T1W, T2W, mDIXON and DWI. The image resolution was larger than 512x512 pixel. T2W sagittal images were finally collected for the followed image processing.

### 2.3 MRI image instance segmentation

MRI image was processed based on the instance segmentation model which has been published by our team (16).

MRI images are characterized by the presence of offset fields, low contrast and blurred uterine tissue boundaries, which increase difficulty in AI automatic segmentation.

In order to solve this problem, adaptive histogram equalization was used to adjust the contrast between uterine tissues, especially for uterine fibroids and uterine wall with similar in signal intensity. The N4ITK method was used to correct the offset field problem, and the Z-Score method was used to normalize the MRI images to the same range. Manual intervention was strictly prohibited except for initial DICOM-to-NIfTI conversion using dcm2niix (v1.0.20220720).

A specialized network architecture was meticulously crafted for image processing in this study. Initially, the high-resolution network v2p (HRNetv2p) was employed for high-resolution feature extraction and multi-scale feature fusion operations within the backbone section. This strategic utilization aimed to ensure effective extraction of small-scale targets in the uterine region. To address the challenge posed by diverse organ shapes, deformable convolutional networks (DCN) were incorporated. DCN facilitated the extraction of authentic feature information from varied shapes, mitigating the loss of shape-specific information.

Furthermore, the convolutional block attention module (CBAM) played a crucial role in feature extraction. Its function included filtering out irrelevant and interfering feature information while enhancing the feature expression capability of the AI model. To aid in target localization, an anchor-based approach was implemented, contributing to the overall effectiveness of the image processing methodology.

The dimensions of fibroids, uterine walls, and uterine cavities within the uterine region exhibit considerable variability, rendering conventional size settings inadequate. In our previous work, distribution statistics were conducted on the length, width, and aspect ratio of the minimum peripheral bounding box of the target within our dataset. This statistical analysis served as a reference for MR image processing. The K-Means clustering method was applied to determine the number of clusters in the target bounding box, thereby determining the appropriate box size. This approach was simultaneously employed across different feature layers to enhance the detection of small-scale targets in the shallow layer and large-scale targets in the deep layer.

In the segmentation task, the PointRend module was introduced to optimize segmentation edges iteratively between adjacent targets. This iterative segmentation strategy effectively reduced jaggies and rough edges, resulting in smoother and more detailed edges for various objects within the uterine region. Given that the model encompasses multiple subtasks, the loss function comprises several components. The classification loss function evaluates the accuracy of target classification using cross-entropy loss. The bounding box loss function assesses the accuracy of target localization through smooth L1. Additionally, the segmentation loss function consists of two parts, namely Coarse mask head and mask point rend, primarily calculated through binary cross-entropy loss.

As the gold standard used as a reference for segmentation, the board-certified radiologists (10+ years in gynecological MRI) independently annotated all structures using 3D Slicer (v5.2.1): 1.Fibroids: Manual contouring on T2WI axial sequences. 2.Uterine wall: Semi-automated segmentation with level-set refinement. 3.Cavity: Threshold-based segmentation (intensity >200 on T2WI). Inter-rater reliability was excellent (Dice similarity coefficient [DSC]: 0.92 ± 0.03 for fibroids). Final ground truth was generated via STAPLE algorithm.

### 2.4 Measurement methods

The clinical data, including age, weight, height, BMI, pregnancy times, labor times, abortion times, clinical symptoms, operation time, blood loss, reproductive hormone level, and postoperative recovery, such as restoration of intestinal function, body temperature, were analyzed in this research. The size, type and position of uterine fibroids were measured using MRI and AI models we built. Time for separating adhesions and removing fibroid specimens from the abdominal cavity was not included in the operation time.

### 2.5 Statistical analysis

Statistical analysis was realized using the SPSS software (version 26.0, SPSS Inc., Chicago, IL, USA). Quantitative data that conform to normal distribution were expressed as mean ± standard deviation (SD). Comparisons between the data were performed with t test. Quantitative data that do not fit a normal distribution are expressed as percentiles. Comparisons between the data were performed with Mann-Whitney U test. Qualitative data were expressed as number and percentage. And chi-square test was performed to analyze the difference of the two groups. Probability values of p<0.05 were considered significant.

## 3 Results

### 3.1 General clinical characteristics

Participants were divided equally into two groups based on the presence or absence of AI involvement, each containing 60 patients. **Table 1** presented the clinical characteristics. No significant differences were found in age, weight, height, BMI, times of pregnancy and childbirth, symptoms including menstrual variation, urinary system compression such as frequent urination, urinary retention, dysuria, and hydronephrosis, digestive system compression such as constipation, anemic, abdominal pain, and reproductive hormone between the two groups(p>0.05). Besides, no significant difference was found in the fibroid size(6.67(6.00-8.00)cm vs. 7.00(6.00-8.00)cm, p=0.96).

**Table 1:** General clinical characteristics.

	Total	MRI	MRI-AI	p-value
patient (n)	120	60	60	
Age	39 (35-42)	39 (36-42)	39 (35-41)	0.38
height (cm)	161 (160-165)	161 (158-165)	162 (160-164)	0.8
weight (kg)	61 (55-69)	51.50 (55.25-69.00)	61.00 (55.00-69.75)	0.77
BMI (kg/m2)	23.88 (21.20-26.49)	23.79 (20.99-26.27)	24.33 (21.56-26.56)	0.65
pregnance	2 (1-3)	2 (1-3)	2 (1-2.75)	0.29
birth (n)	1 (0-1)	1 (1-1)	1 (0-1)	0.68
Vaginal delivery	0 (0-1)	0 (0-1)	0 (0-1)	0.45
cesarean section	0 (0-1)	0 (0-1)	0 (0-1)	0.80
abortion	1 (0-2)	1 (0-2)	1 (0-1)	0.43
intermenstrual bleeding (n)	7 [5.83]	5 [0.12]	2 [3.33]	0.43
menstrual variation (n)	24 [20.00]	13 [21.67]	11 [18.33]	0.82
menstrual cycle change (n)	21 [17.5]	13 [21.67]	8 [13.33]	0.34
increased menstrual flow (n)	39 [32.50]	21 [35.00]	18 [30.00]	0.70
changes in dysmenorrhea (n)	3 [2.50]	1 [1.67]	2 [3.33]	1.00
abnormal leukorrhea (n)	1 [0.83]	1 [1.67]	0 [0.00]	1.00
frequent urination (n)	44 [36.67]	20 [33.33]	24 [40.00]	0.57
urine retention (n)	1 [0.83]	0 [0.00]	1 [1.67]	1.00
difficulty urinating (n)	2 [1.67]	2 [3.33]	0 [0.00]	0.50
fluid retention in the kidneys (n)	1 [0.83]	1 [1.67]	0 [0.00]	1.00
difficulty in defecating (n)	5 [4.17]	4 [6.67]	1 [1.67]	0.36
lower limb edema (n)	1 [0.83]	1 [1.67]	0 [0.00]	1.00
abdominal pain (n)	18 [15.00]	9 [15.00]	9 [15.00]	1.00
spin (n)	11 [9.17]	8 [13.33]	3 [5.00]	0.20
anemia (n)	42 [35.00]	20 [33.33]	22 [36.67]	0.85
mild anemia (n)	30 [25.00]	15 [25.00]	15 [25.00]	1.00
moderate anemia (n)	10 [8.33]	3 [5.00]	7 [11.67]	0.32
severe anemia (n)	2 [1.67]	2 [3.33]	0 [0.00]	0.50
FSH	6.02 (5.13-7.10)	5.84 (4.81-7.17)	6.15 (5.23-7.07)	0.4
LH	5.28 (3.96-6.93)	5.00 (3.67-6.28)	5.67 (4.09-7.06)	0.18
P	0.62 (0.51-0.76)	0.61 (0.49-0.80)	0.63 (0.52-0.72)	0.92
E2	90.12 (80.41-96.02)	90.14 (80.55-96.44)	90.12 (80.34-95.14)	0.96
T	0.36 (0.23-0.48)	0.37 (0.24-0.50)	0.35 (0.22-0.42)	0.28
PRL	9.81 (7.52-12.57)	9.17 (7.36-12.97)	10.50 (7.75-12.40)	0.42
fibroid size (cm)	7.00 (6.00-8.00)	6.67 (6.00-8.00)	7.00 (6.00-8.00)	0.96

BMI, Body Mass Index; FSH, Follicle-Stimulating Hormone; LH, Luteinizing Hormone; PRL, Prolactin; E2, Estradiol; T, Testosterone.

Data presented as median (IQR) for continuous variables; n[%] for categorical variables.

### 3.2 MRI image instance segmentation

**Figure 2:**
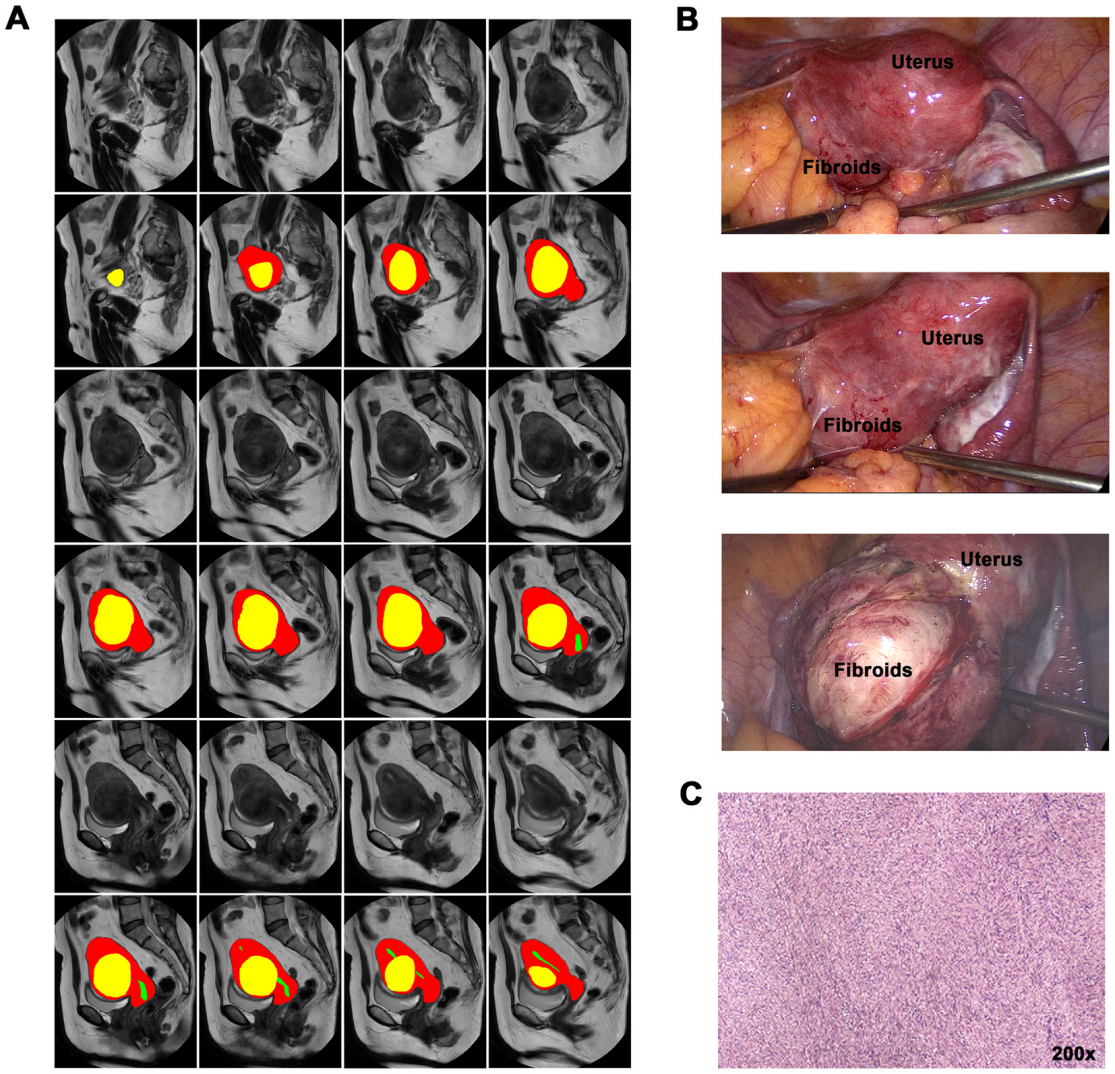
AI instance segmentation results and clinical correlation. **(A)** Axial T2-weighted MRI with AI segmentation overlay: Yellow: Uterine fibroid. Green: Uterine cavity. Red: Uterine wall. **(B)** Intraoperative laparoscopic view corresponding to **(A)**, showing fibroid and uterus. **(C)** Postoperative pathology specimen confirming leiomyoma diagnosis.

**Figure 2** showed the results of the instance segmentation of AI model. Inference masks were covered on the original MRI images, representing uterine fibroids(yellow), uterine cavity(green) and uterine wall(red). **Figure 2A** represents original MRI image and the inference masks generated by our AI model. **Figure 2B** demonstrates the intraoperative view and **Figure 2C** shows the postoperative pathology.

### 3.3 Operative outcomes

**Figure 3:**
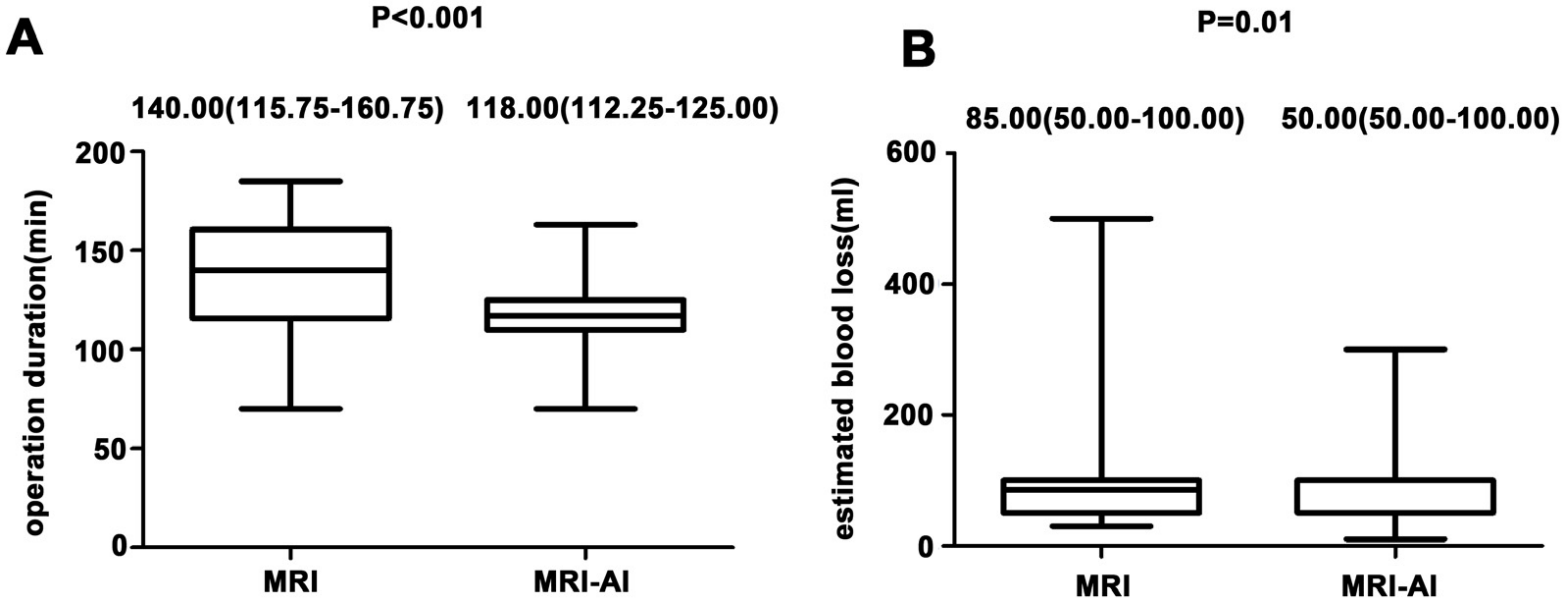
**(A)** Differences in operation time in both group. **(B)** Differences in blood loss in both groups.

The operative outcomes in group MRI and group MRI-AI were both presented in **Table 2**. No significant differences were found in perioperative hemoglobin changes, postoperative fever, postoperative abdominal drainage within 24 hours and hospitalization days(p>0.05). Meanwhile, the differences in operation time(140.00(115.75-160.75)min vs. 118.00(112.25-125.00)min, p<0.001), proportion of patients whose surgery lasted no less than 150 minutes(27[45.00%] vs. 4[6.67%],p<0.001), blood loss(85.00(50.00-100.00)ml vs. 50.00(50.00-100.00)ml, p=0.01), and the happen of first flatus within 24 hours after surgery(15[25.00%] vs. 29[48.33%], p=0.01) were found to be statistically significant between the two groups. And the differences were reemphasized in the **Figure 3**. **Figure 3A** showed the differences in operation time and **Figure 3B** showed the differences in blood loss.

**Table 2:** Operative outcomes.

	Total	MRI	MRI-AI	p-value
operation duration (min)	123.50 (113.00-149.00)	140.00 (115.75-160.75)	118.00 (112.25-125.00)	<0.001
operation duration≥150min (n)	31 [25.83]	27 [45.00]	4 [6.67]	<0.001
blood loss (ml)	50.00 (50.00-100.00)	85.00 (50.00-100.00)	50.00 (50.00-100.00)	0.01
blood loss≥150ml (n)	20 [16.67]	13 [21.67]	7 [11.67]	0.22
preoperative hemoglobin (g/l)	126.5 (118-134.75)	126.00 (113.50-134.75)	129.00 (121.00-135.50)	0.17
postoperative hemoglobin (g/l)	110.00 (102.00-119.75)	108.00 (96.00-119.00)	114.00 (103.50-121.00)	0.11
perioperative hemoglobin changes (g/l)	15.68 ± 9.81	15.77 ± 10.60	15.58 ± 9.05	0.92
postoperative abdominal drainage (ml)	150 (90-167.50)	150.00 (92.50-170.00)	140.00 (80.00-160.00)	0.73
first flatus within 24 hours (n)	44 [36.67]	15 [25.00]	29 [48.33]	0.01
postoperative fever (n)	93 [77.50]	49 [81.67]	44 [73.33]	0.38
(body) temperature≥38.5°C	8 [6.67]	3 [5.00]	5 [8.33]	0.72
Post-operative hospitalization days (day)	5 (5-6)	5 (5-6)	5 (5-6)	0.98

## 4 Discussion

In the ongoing pursuit of minimizing trauma and enhancing postoperative recovery, numerous innovative technologies have been integrated into laparoscopic surgery. In this study, we introduced a groundbreaking artificial intelligence (AI) automatic instance segmentation model specifically designed for magnetic resonance images (16). The implementation of this AI technology has yielded notable improvements in the operation time, intraoperative blood loss, and postoperative recovery of bowel function. These enhancements can be primarily attributed to the AI technology ‘s capacity to assist gynecologists in the procedure of clinical decision. Throughout the surgery, the AI technology enables gynecologists to discern anatomical relationships with heightened precision, thereby augmenting the efficiency and safety of the surgical procedure.

With a prevalence of uterine fibroids surpassing 70 percent, paper reported that around 200,000 hysterectomies and 30,000 myomectomies are performed annually (17), underscoring the considerable trauma and social burden associated with this disease. In the realm of modern medicine, gynecologists are actively exploring choices to make procedures less invasive, swifter, safer, and to facilitate patients ‘ postoperative recovery.

Laparoscopic myomectomy (LM) is increasingly being adopted in the treatment of uterine fibroids (18). Recent systematic reviews highlight that 34% of LM conversions to laparotomy stem from inadequate fibroid localization, particularly in anatomically complex cases (33). The significance of adequate detection and localization of uterine fibroids cannot be overstated. Despite potentially longer procedural duration than open myomectomy, LM is preferred due to its notable advantages, including shorter hospital stays, fewer sutures, smaller incisions, and improved pain management (19, 20). However, challenges such as postoperative recurrence and intraoperative bleeding persist in LM (21). Yoo EH et al. reported recurrence rates of 11.7%, 36.1%, 52.9%, and 84.4% at 1, 3, 5, and 8 years after LM, respectively, with a reoperation probability of 6.7% after five years and 16% after eight years (22). Compared to open myomectomy, LM presents difficulties in detecting small fibroids deep within the myometrium through palpation of the uterine corpus, particularly in cases of multiple fibroids, leading to potential omissions. Additionally, LM may hinder the complete removal of as many fibroids as possible intraoperatively due to existing limitations of diagnosis in accurately determining the locations of small or multiple fibroids. The integration of preoperative magnetic resonance imaging proves timely in addressing the need of detection and localization of uterine fibroids.

Addressing complications, Paul GP et al. conducted a study encompassing 1001 cases, analyzing complications of LM performed by the same surgeon (23). In this study, the mean intraoperative blood loss was 248 ml. It is noteworthy that an increase in intraoperative bleeding is correspondingly associated with a prolonged procedure duration, and conversely, a lengthening of the procedure duration tends to increase intraoperative bleeding. Instances of conversion to hysterectomy have been reported in approximately 0.37%-2.7% of cases in situations of excessive bleeding (20, 24). Such conditions can inflict additional trauma on the patient and impede postoperative recovery.

The adoption of Enhanced Recovery After Surgery (ERAS) in gynecological surgery has gained widespread emphasize. ERAS facilitates accelerated postoperative recovery, reduced hospital stays, enhanced patient satisfaction, and decreased healthcare costs. However, ERAS may not place too much emphasis on the operator or the procedural completion. Christopher G. Smith et al. discovered that patients with at least one surgical complication were ten times more likely to experience a prolonged postoperative hospital stay (25). Shortening the duration of laparoscopic surgery and minimizing bleeding can lead to a reduction in intraoperative anesthetic dose, carbon dioxide intake, and fluid intake, thereby facilitating adherence to ERAS principles.

To achieve these goals, gynecologists are continually upgrading their laparoscopic equipment and honing their surgical skills. Notably, laparoendoscopic single-site (LESS) surgery and robotic-assisted laparoendoscopic single-site (RA-LESS) surgery have gained widespread use in various gynecologic procedures, including myomectomy (26). Both LESS and RA-LESS myomectomy methods reduce trauma to the patient ‘s abdominal wall, demonstrating potential advantages in terms of fewer postoperative complications and improved aesthetics (27, 28). However, it is essential to acknowledge that these surgeries entail a steep learning curve, and most hospitals in China lack the requisite equipment or physician resources for their implementation, rendering these techniques currently unavailable to the majority of patients. Furthermore, several retrospective studies have indicated no significant differences between conventional LESS and RA-LESS and standard laparoscopic myomectomy in terms of operative time, intraoperative blood loss, recovery time, length of hospital stay, and postoperative complications (29, 30).

Artificial intelligence(AI) is expected to play a crucial role. Medical image processing techniques have undergone significant advancements in recent years, attributed largely to the emergence of AI, particularly deep learning technology. Deep learning exhibits the capacity to automatically discern the presence of specific anatomical structures within laparoscopic images by detecting and recognizing the ongoing procedure (31, 32). Its inherent capability to autonomously localize and highlight crucial anatomical structures during surgery serves to enhance overall surgical safety. Sabrina Madad Zadeh et al. contributed a dataset of laparoscopic gynecological images with meticulously labeled anatomical structures and instrumentation tools (7). While this dataset facilitated semantic segmentation of laparoscopic images for surgical guidance, its practical clinical application, particularly in laparoscopic myomectomy, presents obvious limitations. While semantic segmentation aids in recognizing anatomical structures, it is evident that this approach has limited utility in myomectomy. Qualified gynecologists can readily differentiate between organs during surgery, and for myomectomy, it is crucial to determine the relationship between the uterine fibroid, uterine wall, and uterine cavity. In this context, instance segmentation techniques prove more advantageous than semantic segmentation techniques. Carmelo Militello et al. introduced a novel segmentation method for the automatic segmentation of the uterus and fibroids using fuzzy C-Means clustering and an iterative optimization threshold selection algorithm (14). While effective in objectively assessing the magnetic resonance-guided focused ultrasound therapy, this technique only isolates the fibroids from the uterus, overlooking the essential uterine cavity. This might be attributed to the lower demand on uterine cavity information in high-intensity focused ultrasound (HIFU) for fibroids compared to LM.

Nicolas Bourdel et al. explored augmented reality during LM, combining preoperative MRI image segmentation, 3D reconstruction, and intraoperative 3D images of organs (9). The study demonstrated potential safety and efficiency benefits. However, the initial step involved manual segmentation of preoperative MRI images, revealing limitations in accuracy and time-consumption. Additionally, the study comprised only three case studies, necessitating further feasibility validation. Yoshifumi Ochi et al. recently reported a case utilizing mixed reality technology during LM (10). Nonetheless, similar to the study by Nicolas Bourdel et al., these studies leave certain limitations unaddressed. Efforts to enhance segmentation accuracy and streamline the application of mixed reality technology in LM are essential areas for further exploration and development.

Our AI-based instance segmentation approach addresses critical limitations of prior methods. Unlike augmented reality systems that rely on manual MRI segmentation and 3D reconstruction—processes prone to human error and time delays—our model automates segmentation with higher accuracy, reducing preoperative preparation time. Semantic segmentation frameworks lack the granularity to distinguish individual fibroids, whereas our instance segmentation preserves topological relationships between multiple fibroids and critical structures like the uterine cavity. This capability is absent in HIFU-focused methods, which exclude uterine cavity data. By integrating cavity information, our system enables surgeons to avoid inadvertent damage to the endometrium, a risk inherent in LM. Compared to mixed reality systems tested in small case studies, our AI demonstrated scalability in a cohort of 120 patients, with results validated across multiple institutions. These advancements directly translate to superior clinical efficiency: our model reduced operative time compared to non-AI-assisted LM.

The clinical impact of our AI system is multifold. First, the reduction in intraoperative blood loss lowers transfusion needs. Second, shorter operative times (113 ± 28 minutes vs. 145 ± 35 minutes) reduce anesthesia exposure and hospital resource utilization, aligning with ERAS principles to cut postoperative stays. Third, improved fibroid localization accuracy minimizes residual fibroids, potentially reducing recurrence rates—a critical factor given the 84.4% 8-year recurrence rate. Patient outcomes are further enhanced through minimized collateral tissue damage, which accelerates bowel function recovery and reduces postoperative pain.

Unlike conventional computer vision approaches limited to semantic segmentation, our instance segmentation framework uniquely preserves topological relationships between multiple fibroids - a critical feature for avoiding collateral damage during morcellation. In this study, our team employed a novel instance segmentation model to facilitate automatic preoperative segmentation of MRI images, aiding gynecologists in enhancing awareness of uterine fibroids. This approach demonstrated notable advantages, contributing to expedited procedures, reduced bleeding, and improved postoperative recovery, particularly in terms of the recovery of bowel function. These improvements are attributed to the AI’s ability to preserve topological relationships between fibroids and critical structures, minimizing collateral damage. However, our findings are currently limited to single fibroid type. To ensure broader applicability, we are initiating a multicenter trial to evaluate the system’s performance in complex scenarios, including multifocal and deep intramural fibroids. Challenges such as clinician training and infrastructure compatibility will be addressed through targeted workshops and cloud-based solutions. Future work will also integrate 3D reconstruction to enhance preoperative planning and explore long-term outcomes, including recurrence and fertility rates.

However, it is important to note that only improvements in bowel function recovery have been identified, with no observed optimizations in postoperative fever or hospitalization duration. This lack of optimization can be attributed to the multifaceted nature of factors influencing postoperative recovery, extending beyond procedural duration and intraoperative bleeding.

Furthermore, our study focused specifically on single broad ligament fibroids, and the applicability of the results to cases involving multiple fibroids or different types of fibroids remains to be established. We recognize these limitations and plan to address them comprehensively in our future work. The relatively small sample size and short postoperative observation period further constrain the generalizability of our findings. Long-term aspects of recovery, such as fertility and uterine rupture rates during pregnancy, could not be determined in this study. To address these limitations, we are actively working to expand our case pool and planning to initiate a joint multicenter study to corroborate and extend our findings. While our current study focused on single broad ligament fibroids, we acknowledge the need to validate the model’s efficacy in cases with multiple or deeply embedded fibroids. Our next phase involves a multicenter trial to test the AI system on 200+ patients with diverse fibroid types (submucosal, intramural, subserosal) and quantities.

Our findings redefine preoperative planning standards for complex myomectomy, demonstrating that this AI system reduces operative time and blood loss compared to conventional laparoscopic myomectomy (LM). The system also improves adherence to the ERAS protocol by shortening hospitalization. These results suggest that AI-assisted LM could become the standard of care for managing broad ligament fibroids, particularly in high-volume centers.

Prior studies have primarily focused on semantic segmentation of generic uterine anatomy or on augmented reality systems requiring manual input. Our work introduces three novel advancements: 1.An instance segmentation framework specifically tailored to the unique retroperitoneal anatomy of broad ligament fibroids. 2.Automated MRI-to-laparoscopy coordinate mapping, which eliminates dependency on radiologists. 3.Quantitative evidence demonstrating the superiority of AI over both conventional laparoscopic myomectomy (LM) and mixed reality systems in controlling bleeding.

These innovations address a critical gap in the management of broad ligament fibroids, where traditional imaging fails to adequately visualize parametrial interfaces. Moreover, the automated pipeline requires no specialized radiologist input, making advanced planning accessible in resource-limited settings—contrasting sharply with augmented reality systems that rely on expert segmentation.

Additionally, the segmentation results in our study were confined to 2D MRI images, which may not provide sufficient detail to accurately discern the number and location of fibroids. To overcome this limitation, we have initiated a study on preoperative 3D reconstruction based on automatic instance segmentation, yielding partial results. Our ongoing research endeavors will encompass methodological refinements, seamless clinical integration, and robust validation. The role of artificial intelligence in optimizing laparoscopic myomectomy will be a key focus in our future research initiatives.

We recognize potential barriers, such as clinician acceptance and institutional readiness. To mitigate this, we plan to: 1.Conduct hands-on workshops for surgeons to familiarize them with AI tools. 2.Collaborate with hospitals to standardize MRI protocols for AI compatibility. 3.Address computational infrastructure gaps in resource-limited settings through cloud-based solutions. Future studies will track long-term metrics (e.g., recurrence rates, fertility outcomes) over 5–10 years, as our current observation period was limited to 6 months.

## 5 Conclusion

This study demonstrates that our AI-powered uterine fibroid instance segmentation model, leveraging preoperative MRI, significantly enhances the efficiency of laparoscopic myomectomy (LM) and accelerates postoperative recovery. By automating fibroid localization with high accuracy and reducing operative time and blood loss by, this technology addresses critical challenges in LM, such as incomplete fibroid removal and intraoperative complications.

Future Directions and Applications

Technical Refinements:

Develop 3D reconstruction capabilities to overcome current 2D MRI limitations, enabling precise spatial mapping of fibroids relative to vasculature and the uterine cavity. Optimize the AI algorithm for real-time intraoperative guidance, integrating it with laparoscopic imaging systems to dynamically adjust surgical planning.

Clinical Expansion:

Validate the system in multicenter trials involving complex cases (e.g., multifocal, deep intramural fibroids) and diverse patient populations. Extend the framework to other gynecological procedures, such as endometriosis resection and ovarian cystectomy, where anatomical precision is equally critical.

Implementation Strategies:

Partner with hospitals to standardize AI-compatible MRI protocols and establish cloud-based solutions for resource-limited settings. Conduct surgeon training programs to bridge the gap between AI tool adoption and clinical expertise.

Long-Term Goals:

Investigate the AI system’s impact on fertility outcomes and recurrence rates over 5–10 years, addressing the current short-term follow-up limitation. Explore cost-effectiveness analyses to quantify reductions in healthcare expenditures, particularly in avoiding reoperations.

By prioritizing these steps, our research aims to transition from a proof-of-concept model to a universally accessible tool, revolutionizing minimally invasive gynecologic surgery. This roadmap not only refines the AI’s technical performance but also ensures its seamless integration into clinical workflows, ultimately improving patient care and surgical standards globally.

## Data Availability

The original contributions presented in the study are included in the article/supplementary material. Further inquiries can be directed to the corresponding author/s.
